# Intrinsic dietary fibers and the gut microbiome: Rediscovering the benefits of the plant cell matrix for human health

**DOI:** 10.3389/fimmu.2022.954845

**Published:** 2022-08-18

**Authors:** Marie-Luise Puhlmann, Willem M. de Vos

**Affiliations:** ^1^ Laboratory of Microbiology, Wageningen University & Research, Wageningen, Netherlands; ^2^ Division of Human Nutrition and Health, Wageningen University & Research, Wageningen, Netherlands; ^3^ Human Microbiome Research Program, Faculty of Medicine, University of Helsinki, Helsinki, Finland

**Keywords:** intact plant cells, plant cell wall, minimal processing, colonic microbiota, gut health, short-chain fatty acids (SCFA)

## Abstract

Dietary fibers contribute to structure and storage reserves of plant foods and fundamentally impact human health, partly by involving the intestinal microbiota, notably in the colon. Considerable attention has been given to unraveling the interaction between fiber type and gut microbiota utilization, focusing mainly on single, purified fibers. Studying these fibers in isolation might give us insights into specific fiber effects, but neglects how dietary fibers are consumed daily and impact our digestive tract: as intrinsic structures that include the cell matrix and content of plant tissues. Like our ancestors we consume fibers that are entangled in a complex network of plants cell walls that further encapsulate and shield intra-cellular fibers, such as fructans and other components from immediate breakdown. Hence, the physiological behavior and consequent microbial breakdown of these intrinsic fibers differs from that of single, purified fibers, potentially entailing unexplored health effects. In this mini-review we explain the difference between intrinsic and isolated fibers and discuss their differential impact on digestion. Subsequently, we elaborate on how food processing influences intrinsic fiber structure and summarize available human intervention studies that used intrinsic fibers to assess gut microbiota modulation and related health outcomes. Finally, we explore current research gaps and consequences of the intrinsic plant tissue structure for future research. We postulate that instead of further processing our already (extensively) processed foods to create new products, we should minimize this processing and exploit the intrinsic health benefits that are associated with the original cell matrix of plant tissues.

## Introduction

Human health is substantially influenced by the food we eat. One food component that especially gained attention during recent years is the indigestible backbone of our plant foods: dietary fibers that cannot be directly utilized by our body. Dietary fibers appear to be an all-round talent reducing all-cause-mortality and protecting against different types of cancer, type 2 diabetes and cardiovascular diseases (1). The hypothesis that fibers have a crucial place in maintaining human health is not new. Already in the 1960’s, Denis Burkitt developed the dietary fiber hypothesis placing dietary fibers at the origin of numerous diseases occurring in high-income countries with a Western lifestyle (2, 3). At that time, many beneficial effects of fibers were linked to gut microbiota-independent effects. These were based on physicochemical properties of fibers, like retaining water, which increases stool bulk and speeds up transit time, or bile-acid binding, which reduces cholesterol levels (2). Gut microbiota-dependent health benefits of fibers where hypothesized, but could not be determined as the present culture-independent high-throughput tools and mechanistic insights were lacking (2, 4, 5). This has changed during the last 25 years (3). As dietary fibers are not broken down by human endogenous enzymes these are passed down to the lower gut where they are utilized by the gut microbiota, mainly consisting of bacteria residing in the colon (6). The gut bacteria thereby produce various metabolites, most importantly short-chain fatty acids (SCFA) that mediate some of the microbiota-dependent effects of fibers (7–9). These effects can range from locally interacting with epithelial and immune cells affecting barrier function and gut homeostasis (8–10) to peripherally impacting organs either by receptor-binding or by exerting direct effects (7, 11). As a consequence, modulating gut microbiota activity and composition by fiber intake holds potential new therapeutic avenues. In this context, distinct fiber types have been studied to elucidate their specific microbiota-dependent and -independent effects on human health (12). This has led to a continuous effort to further narrow down the underlying fiber-microbiota interactions *in vitro* and to understand the specific molecular make-up of fibers as for instance leading to the immunomodulatory potential of different fiber chain lengths and structural features (13, 14). These efforts, however, follow a reductionist approach that considers fibers as loose entities (15–17). This single-fiber-approach is in line with Western food processing and food design practices but neglects the original form in which fibers are present and consumed during our evolution: as part of whole foods and often only minimally processed.

Here we elaborate on the need to change our understanding of dietary fibers to unlock their full potential to modulate gut microbiota in relation to human health. We explain how dietary fibers exist in nature and discuss how this holistic view differs from the current approaches. Finally, we explore the existing intervention studies of intrinsic fibers in relation to human health and discuss potential consequences for future research avenues.

## Current view of dietary fibers: single, isolated components and their impact

Dietary fiber is an umbrella term for a group of polymers that are structurally and chemically very different. Numerous sugar molecules, such as glucose, xylose, mannose, galactose, arabinose and rhamnose are linked together by various glycosidic bonds following a specific or random pattern creating linear or branched structures that can be further decorated with phenolic acids or linked to other compounds (18, 19). For instance, inulin and starch consist of linear repetitions of a single molecule (fructose and glucose monomers, respectively). In contrast, rhamnogalacturonan – a type of pectin – is a highly branched molecule with various side-chains of different monomers (20). What makes them dietary fibers is the common trait of their bonds not being broken by human endogenous enzymes.

The enormous variation in fiber structures brings along physicochemical properties that are often classified into “soluble versus insoluble”. This classification, despite being widely used, is only based on fiber content analysis of foods rather than functional behavior of the fibers in the human gut (21–25). Functional fiber properties are therefore better described in terms like bulking, viscosity and fermentability (23). Fermentation of the different fibers by the gut microbiota requires microbes to have at their disposal a set of enzymes that can break down the specific chemical linkages and types of molecules (6, 26). An often-observed feature of microbial communities is functional redundancy, meaning that taxonomically different members of the gut microbiota can perform similar functions, e.g. break down the same type of fiber (6, 27, 28). Microbes within the gut microbiota community can assist each other *via* cross-feeding, with primary degraders cleaving polymers into smaller compounds that can be further broken down by others and finally be converted into SCFA (6, 28–30). Since the vast majority of the gut microbiota consists of mainly anaerobic bacteria, these are the main contributors of the fiber degradation, occasionally supported by the action of methanogens that convert generated hydrogen into methane (29, 31). While some of these bacteria are adapted to metabolize a wide range of polymers, others appear to be more specialized (6, 28). Moreover, specific bacteria have been coined to be so-called “keystone” species as they are unique players in the metabolic networks for the breakdown of compounds or the production of specific metabolites (6).

Multiple intervention studies with isolated, single fibers have been reviewed elsewhere (32–38). The emerging picture is that i) a range of different fibers are able to stimulate a more diverse range of gut bacteria (39), and ii) chemically and structurally complex fibers can be used to specifically target bacteria relevant for human health (40–42). Using this approach, efforts have been made to define differences in the fine structure of fibers and relate these to the specific gut microbiota response and health outcomes (12, 39, 43). For instance, in a recent human trial wheat bread was enriched with a variety of fibers like wheat dextrin, micronized wheat bran, oat flakes and bran, inulin, locust bean gum and pectin, which to some degree decreased cholesterol, insulin and HOMA-IR levels and these changes were linked to an increase in the gut microbiota able to break glycosidic bonds (44). Similarly, another recent human trial in overweight individuals linked the gut microbiota’s ability to ferment arabinoxylan but not crystalline cellulose to an observed increase in satiety (45). Moreover, *in vitro* assessment of different fast-fermentable fiber types indicated a delayed fermentation rate for some fibers when presented in a mix instead of alone (46). A delayed fermentation rate is hypothesized but not yet shown to be a desired feature for the therapeutic application of fibers in the treatment of irritable bowel syndrome (23). All these studies have as a common principle that they rely on single fibers or fiber extracts that have been isolated from the plant tissue they originate from. Understanding the isolated effects of these single fiber types has its place in determining trophic chain interactions between microbes (29) and to unravel underlying mechanisms for specific fiber applications in e.g. the medical field (47). However, looking at fibers as isolated components overlooks one aspect of dietary fibers: how we eat them.

## Intrinsic fibers: complexly intertwined three-dimensional structures

When we discuss dietary fibers, we mainly think of them as single, loose compounds. However, this contrasts with their “natural form” - the form already recognized by Burkitt to entail the crucial health benefits of fibers. The bulk of fibers we eat are not single, isolated fibers, but part of plant foods like vegetables, fruits, seeds, nuts, legumes and grains (22, 48–51). We consume the tissues of these plants, which are made up from a matrix of plant cells (52). The backbone of this matrix are dietary fibers that are complexly intertwined into a three-dimensional network creating plant cell walls (52). These plant cells can further encapsulate other fibers that are stored in the vacuoles, which are either fructans or starch serving the plant as reserve for growth (53, 54). During recent years, awareness has risen that the existence of this three-dimensional plant cell matrix has important consequences for digestibility and health while very likely exerting different effects than the single, isolated fibers. For this purpose, a distinction was made between isolated fibers and fibers in their natural, three-dimensional form. The latter fibers were termed “intrinsic fibers” (22) referring to these as being an intrinsic part of the plant cell wall.

### Make-up of intrinsic fibers

**Figure 1:**
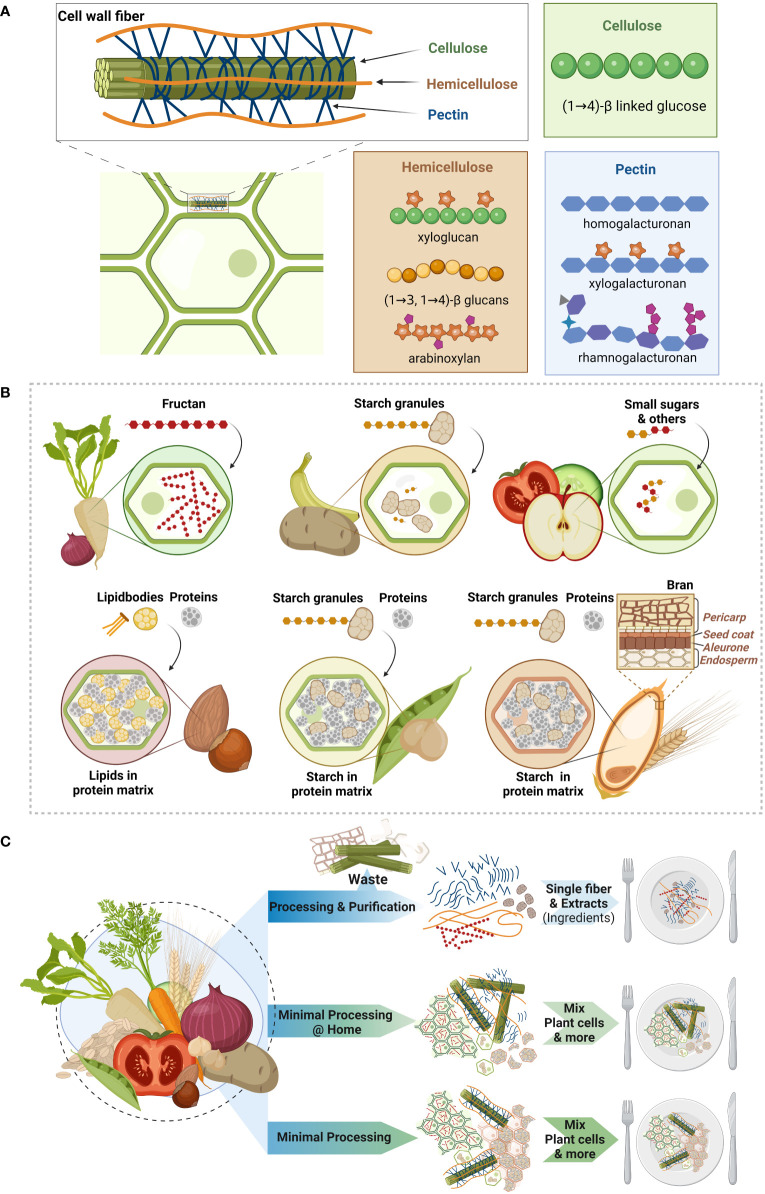
Intrinsic make-up of dietary fibers in plant foods **(A)** Dietary fibers are the backbone of plant foods that we consume, like grains, legumes, fruits, vegetables, nuts and seeds. The intrinsic make-up of plant tissue is created by the plant cell wall fibers cellulose, hemicellulose and pectin. Cellulose (green) forms fibrils, which are stabilized by hemicellulose (orange) and further strengthened by pectin (blue) forming a complexly intertwined three-dimensional structure. While cellulose is a structurally simple molecule made of glucose molecules that are β(1→4) linked, hemicellulose and pectin are two groups of structurally very diverse cell wall fibers. An abundant hemicellulose in dicot plants (like legumes) is xyloglucan, while monocot plants (grains) have higher amounts of mixed linkage β-glucans (e.g. oats) and arabinoxylans (e.g. wheat). Pectin can either consist of linear chains of monomers (homogalacturonan), that can be esterified and further decorated with other sugars (xylogalacturonan) or of several molecules with side-chains made of other compounds (rhamnogalacturonans). **(B)** The complex cell wall fiber structure encapsulates various other nutrients in the plant vacuole like storage carbohydrates, which can be either fructan – a dietary fiber with a fructose backbone - or starch. Starch is stored in starch granules, which are found in vegetables and fruits like potatoes and (unripe) bananas. Other fruits and vegetables’ plant cells contain vacuoles mainly filled with water and other nutrients (e.g. small sugars). Nuts contain lipid bodies that are embedded within the vacuole in a protein matrix, while in legumes starch granules are embedded in the protein matrix. Finally, in contrast to the beforementioned dicot plants, grains, which are monocots, have different plant cell walls but also have starch granules embedded in a protein matrix. This starch-protein matrix is surrounded by the so-called aleurone layer, the seed coat and the pericarp that form together the so called bran. **(C)** Dietary fibers can be processed in various ways that either disintegrate or maintain the three-dimensional plant tissue matrix. Common approaches in the industry are to extract and purify dietary fibers into single fibers creating (new) ingredients that are used to create new food products. Cooking and other domestic processing types maintain the tissue matrix to different extents but can generally provide intact plant cell wall structures. Finally, we propose that similar to domestic processing, plant foods should be minimally processed such that the plant cell wall structure and overall plant tissue matrix are maintained. The latter opens avenues for new, convenient and minimally processed fiber products that exploit the health benefits of the intrinsic plant structure, while aligning with the need for more sustainably produced, plant-based food solutions. Created with BioRender.com.

The make-up of plant cells follows a common principle, which consists of three main building blocks: cellulose, hemicellulose and pectin (**Figure 1A**) (52, 55). Cellulose is considered as basic scaffold of the cell wall, being a linear polymer of β(1→4) linked glucose units that align into rigid microfibrils. These microfibrils are reinforced by hemicellulose (**Figure 1A**), which is a group of structurally very diverse polymers with either glucose-xylose (xyloglucans), a glucose (mixed-linkage glucans) or xylose (xylan) or mannan backbones (24, 56). To these backbones other sugar molecules and also phenolic acids can be linked, such as the hemicellulose type arabinoxylan (56, 57). Finally, pectin serves like a filler giving the cellulose-hemicellulose scaffold stability, controlling permeability and together creating a matrix that can retain water (**Figure 1A**) (55, 58). Similarly to hemicellulose, also pectin is a group of structurally diverse polymers, which have a linear galacturonic acid backbone (homogalacturonans) or a galacturonic acid and rhamnose backbone (rhamnogalacturonans) that can be further decorated with side-chains forming complexly branched polymers (**Figure 1A**) (20, 24, 59). It is essential to understand that these different polymers are physically entangled and partly also chemically bound to each other, despite the fact that their precise organization is still not fully understood (55). These complex structures form together three-dimensional plant cells that are glued together by pectin, which fills the intercellular space between connecting plant cells (55). Besides the fiber polymers, plant cell walls also contain small amounts of proteins (arabinogalactan proteins) and minerals (52, 55). Finally, certain specialized cell-types are reinforced with lignin, which is a dietary fiber consisting not of sugars but of different types of phenolic phytochemicals that are complexly linked (52, 55). While all plants share these common aspects of the plant cell wall, their make-up differs depending on the type of plant (e.g. monocots versus dicots; see below), its maturation or ripeness and especially food processing (18, 52, 58). Even within plants, differences in cell types exist between the tissue we consume (such as leaf, root, fruit, stem, seed) and within these tissues (52, 56).

As hemicellulose and pectin are groups of very heterogenous polymers, the specific types of these fibers that are present in the plant cell walls distinguish different plant types (52, 58). Vegetables, fruits, nuts, seeds and legumes belong to the dicots, that have so-called type I cell walls, while grains are monocots having type II cell walls. Type I cell walls of dicots contain xyloglucan as the most abundant hemicellulose and the hemi-/cellulose network is further stabilized by pectin, which has been reported to make up a third of the cell wall weight (18, 52). In contrast type II cell walls of monocots, have no or very low amounts of pectin and the abundant hemicelluloses are β-glucan and arabinoxylan (52, 58). Rice is one of the grains that contains some pectin, while oats and barley are particularly rich in β-glucan, and wheat, maize and rye in arabinoxylan (57). Another distinguishing feature of grains is the coating that covers the grain kernel. This outer coating consists of several layers known as the aleurone layer, the seed coat and the pericarp, which together form what we know as “bran” (see **Figure 1B**) (60). Bran is therefore not a homogenous compound but contains different cell types that have a different ratio of hemicellulose types than the starchy endosperm (57).

The three-dimensional capsules that are created by the cell wall fibers contain the plant cell with its vacuole in which other nutrients like lipids, proteins and carbohydrates as well as phytochemicals are stored (**Figure 1B**) (53). Depending on the plant type, the encapsulated contents can profoundly differ. For instance, plants store reserve carbohydrates in their vacuoles. These can either be fructans (54), or starch, which is stored in the form of starch granules (**Figure 1B**). While fructans classify as dietary fibers as their glycosidic bonds cannot be broken down by human endogenous enzymes, most types of starch can be digested with some resistant to digestion due to their physical state (resistant starch). Many vegetables and fruits have plant cells whose vacuoles are filled with mainly water, which maintains the internal pressure (turgor) and keeps an open cell structure that provides the food product its characteristic crispiness/crunchiness (**Figure 1B**). The vacuoles also contain other small molecules, such as sugars and phytochemicals (53). In legumes and nuts the vacuoles are filled with a protein matrix, embedding starch granules and lipid bodies, respectively (**Figure 1B**). Finally, the starch granules in grains are also embedded in a protein matrix within the cell walls (**Figure 1B**).

The three-dimensional make-up of plant cells, the physical and chemical entanglement and the encapsulation of other fibers and nutrients have important consequences for the gut microbes’ ability to access the individual fibers. Food processing can substantially influence and destroy these intrinsic structures and is used to extract, isolate and purify single fibers. Moreover, in contrast to these isolated fibers, during upper gut digestion the cell wall fibers do not simply dissolve and become freely available as isolated components but remain to a considerable extent within their intertwined matrix. Hence, the make-up of the plant food cell matrix, food processing and digestion influence how intrinsic dietary fibers are altered in the gut and fermented by the gut microbiota.

### Food processing impacts the intrinsic fiber make-up

Whether a fiber can be classified as intrinsic fiber and how further digestive processes influence its digestive fate depends largely on its processing. As intrinsic fibers are an intrinsic part of the plant cell wall whole, raw foods, like unprocessed apples, bananas, cucumbers, tomatoes etc. contain by definition intrinsic fibers. However, any form of processing - industrial as well as domestic - can alter this three-dimensional structure (19, 24, 52, 58, 61, 62) and whether a fiber is still an intrinsic fiber needs careful checking of the applied methodology.

In general, food processing has as aim to preserve foods, increase digestibility or extract compounds that can be used to create new foodstuffs. To do so, a plethora of techniques can be applied which include mechanical (physical), chemical, thermal and enzymatic processing as well as high pressure, ultrasound and microwave technologies (61, 63). While industrial processing often aims at extracting single fibers for specific food applications, domestic processing is usually gentle and does not fully disintegrate the intrinsic fiber structures (**Figure 1C**). The effects of different processing techniques on the physicochemical properties (64) of fibers as well as cell wall integrity and overall cell structure has been reviewed by others (19, 24, 52, 58). Hence, here we give only a small overview of these techniques to provide a basic understanding of their consequences for intrinsic fibers.

Possibly the most gentle food processing techniques in terms of safeguarding the overall intrinsic tissue structure are drying, freezing and freeze-drying, which aim to conserve food products. While drying leads to shrinkage of the cell tissue, freezing and freeze-drying maintain the open cell structure, but the respective plastic changes of drying and water crystals formed during freezing can induce cracks in the cell wall (65). However, the extent of the caused damage depends on the applied conditions. Consequently, despite cell wall cracks the overall tissue is largely maintained reducing the possible release of intracellular components. Similarly, hydrothermal processing can maintain the overall tissue structure, while inducing more profound damage to cell walls mainly by affecting pectin (24). Boiling or steaming can result in swelling of cell walls and dissolution of pectin (24, 52). When pectin leaks out of the intercellular space it weakens the overall cohesion between cells. This can then lead to separation of plant cells, but also to cell wall rupture. Moreover, leaking of pectin increases the space between the fibers within cell walls, which is called cell wall porosity (24, 52, 58). Dry thermal treatments like baking, roasting or popping are more violent processing types, fundamentally damaging the cell wall, but can also maintain overall tissue structure (52).

Mechanical (physical) destruction, like milling/grinding, generally break cell walls releasing the encapsulated contents (52, 58, 66). The finer the particle size, the less likely it is that whole plant cells are still present and the more likely that parts of the three-dimensional organization of cell walls have undergone physical destruction. Fine milling of the starchy endosperm of grains for instance leads to complete disruption of plant cells, breaking the protein-starch matrix and releasing the enclosed starch granules (**Figure 1B**) (19). Similarly, the complex structure of bran can be substantially destructed with fine milling (49). In this context we want to highlight that despite the suggestive name, whole grain products do not necessarily provide intrinsic fibers (35, 67). The reason is that for the production of whole grain products, first the components bran, germ and the starchy endosperm are separated and each of these components is milled (25, 67). After processing, these fractions are reconstituted together, which allows the product to be defined as whole grain (67). Hence, when we read about whole grain products, we refer to the processed and reconstituted fractions of the whole grain instead of the intact grains (67–69). The intact cells in grains have, however, specific effects on starch accessibility and the gelatinization during heating and thereby impact whether starch escapes upper gut digestion becoming available to the colonic microbiota (52, 70).

In summary, mechanical processing, either domestic or industrial, has major detrimental effects on the intrinsic fiber structure and for instance, dried, pulverized fibers likely do not behave like dried fibers that have undergone minor mechanical destruction. Nevertheless, in these powdered preparations, cell-wall parts might still be present, but their particle size might be very small, increasing the accessibility for gut bacteria. Finally, extrusion, a processing technique where food is subjected to high pressure, high shear and high temperature, is one of the most violent processing techniques (62) as it can completely degrade even the rigid cellulose fibrils (71).

In order to produce single, isolated fibers further extensive extraction techniques are needed that separate the specific fibers from the three-dimensional plant cell matrix (61, 63). Some of these fibers are water-extractable like certain arabinoxylans, which are hence termed “soluble”, while others need the application of enzymatic, gravitational and/or chemical treatments in order to be extracted (57, 63). During these extensive extraction processes, a variety of waste streams are created that might still contain valuable residual fibers and intracellular components. However, the integrity of these structures depends on the harshness of the applied processing. Therefore, fiber products that are based on waste streams might or might not contribute intrinsic fibers. While in some cases the waste streams can be valorized (72, 73), in many cases the production of fibers have a considerable carbon foot print and do not contribute to a circular economy (61, 74).

It is crucial to realize that when we investigate the isolated behavior of single dietary fibers, we in fact study fibers that have undergone extensive processing in order to be released, separated and purified from plant cells (61). It is highly doubtable, that the effects and behaviors of these isolated fibers sufficiently reflect that of intrinsic fibers (52). To illustrate this, we will below describe the impact of intrinsic fibers on digestibility *in vitro* followed by insights from human interventions.

### Implications of intrinsic fibers on digestion

It is clear that food processing substantially impacts how fibers behave in the gut during digestion (24). While the behavior of isolated, single fibers has extensively been studied, the digestive fate of plant tissues is far less understood. The limited research that assessed plant tissue and single plant cells has focused more on the upper gut mainly relying on *in vitro* models and animal *in vivo* models. There are some studies that assessed human ileostomy effluents (with carrots) (75) or ileal samples (with white beans) (76), but very few studies followed the digestion of intrinsic fibers until the colon (77). Here, we will only highlight the main consequences of upper gut digestion for intrinsic fiber structures and refer the reader to the comprehensive reviews by others (19, 24, 52, 58, 78).

Our general understanding of digestion is the breakdown of food matrices into their building blocks and subsequent absorption. However, when consuming plant food tissues with an intact cell matrix, the constituents will not simply dissolve as they are intertwined within the cell wall and in this form not readily water-soluble under physiological conditions (24, 25). Similarly, encapsulated compounds cannot dissolve directly into the gut environment and will be shielded from immediate digestion, delaying their breakdown. In general, the orogastric processes lead to size reduction of plant tissue fractions, and dissolution of certain water-soluble pectins (52). During chewing, plant tissues are degraded into smaller sizes, which depending on the physical state of the plant type (hard, soft), ripeness and preparation methods (like cooking), happens by cell rupture (hard foods) or separation along the cell walls (soft foods) (24, 52, 58). Hence, a mix of differently sized particles containing intact and broken cells and their contents arrives in the stomach. There, further particle breakdown can occur by the mechanical gastric forces. It is generally believed that particles smaller than a few millimeters pass unchanged into the small intestine and, hence, intact plant cells can arrive into the small intestine. Larger structures are retained for further size reduction (called gastric sieving) (58). However, whether this also applies to soft plant tissue is not clear. Pectin can leak from the intercellular spaces (24, 52, 58) and thereby reduce overall cohesion within plant particles. Whether also water-extractable hemicelluloses, like arabinoxylan, can leak out is not known but rather unlikely (24, 57). That means that plant particles with intact cells arrive in the colon. Indeed this has been confirmed from analysis of ileostomy effluents and ileal samples (75, 76). The mixture of broken and intact cells is available to the gastric and small intestinal digestive enzymes to be degraded further, but released fructans and certain resistant starches will resist digestion. Due to pectin leakage cell wall porosity might increase. However, based on available literature, the diffusion of enzymes into intact cells and the diffusion of nutrients out of the cell is believed to be limited by the cell wall pore size (52, 58). Interestingly, the presence of plant cell wall material has been shown to reduce enzymatic breakdown of starch due to adsorption of α-amylase to the plant cell material (19, 52, 58).

In cases of mild food processing, the fractions of intact plant tissue that we swallow likely arrive in the colon in a rather intact state, mainly affected by chewing and dissolution of pectin (**Figure 1C**). Hence, the bacteria in the colon are confronted with a mix of plant tissue particles, intact plant cells and broken cell wall material and its contents. Dissolved fibers, like released fructans or starch (and possibly leaked pectin) can be directly used by the gut bacteria. However, since bacteria are too large to diffuse into intact plant cells, these must spatially interact with the plant tissue, to access intertwined polymers such as pectins and hemicelluloses. All these digestive aspects make it obvious that the breakdown and consequent physiological behavior of intrinsic fibers cannot or can only partly be explained by that of their isolated single counterparts (49, 52).

### Gut microbial intrinsic fiber breakdown

The number of studies that have investigated the microbial breakdown of intrinsic fibers in the colon is impressively low especially regarding human *in vivo* studies. From *in vitro* studies we have a fair understanding of which type of bacteria and bacterial enzymes are generally involved in metabolizing specific isolated fiber types (38, 79). The different enzymes employed by gut bacteria can be classified according to carbohydrate-active enzymes classes (CAZymes; see www.cazy.org (80)), and bacteria differ in the number and type of enzymes they have, as thoroughly reviewed elsewhere (26). The most abundant microbial enzyme class are glycoside hydrolases, cleaving neighboring sugar and non-sugar moieties. Other less abundant enzymes are polysaccharide lyases specialized in the cleavage of uronic acid moieties as present in pectins, and carbohydrate esterases cleaving off esterified groups as present in pectins and hemicelluloses. These enzymes may be assisted by other so-called carbohydrate binding modules or auxiliary activities (80). The bacterial enzymes involved in storage carbohydrate breakdown are relatively well established, like the GH13 subclass for starch and the GH32 subclass for fructan utilization (26), and the discovery of enzyme systems to disintegrate the cell wall fibers pectin (81) and hemicellulose is ongoing (82). However, our knowledge on how gut bacteria interact with the most robust compound of the plant cell wall, the cellulose microfibrils, is still very limited (6, 29). While amorphous cellulose is believed to be partly utilized by employing a complex enzyme system, crystalline cellulose has been reported to be not fermentable (6, 29). Bacteria were found to adhere to cell walls on almond particles (77) and coarse wheat bran (83) recovered from human feces, which indicates that bacteria possibly use the crystalline cellulose backbone for particle adherence (29, 84). A small set of studies tried to identify the bacterial communities colonizing these plant tissue fractions (84–87). Bacteria recovered from plant particles in human feces differed in the detected phyla compared to the liquid fraction (84, 85). Moreover, the type of recovered particles (bran versus resistant starch, differently processed brans) impacted which bacteria adhered to them (86, 87). Based on these observations the breakdown of plant cell walls is believed to be initiated by primary degraders able to interact with the insoluble plant cell material. Subsequently, material is released from cell wall compounds which other bacteria use to cross-feed (6, 29, 84). Some studies have addressed these plant cell-bacteria biofilms and the action of bacterial enzymatic systems that would be needed to degrade cell walls (29, 88, 89). However, how exactly the spatial interaction of these cooperations would look like and how plant cells are exactly opened-up by gut bacteria is unclear. Future research distinguishing between particulate and liquid fecal phases and using imaging techniques combined with identifying bacterial communities will offer exciting insights into these aspects.

It is generally believed that fermentable fibers are readily metabolized in the proximal colon. However, if readily fermentable fibers are not in isolation but either part of plant cell walls or encapsulated by them, the fermentation rate is likely to be slowed down. This has indeed been supported by the results of *in vitro* experiments. For instance, *in vitro* assessment of plant cell-encapsulated cereal starches revealed that microbial enzymatic activity was first directed towards cell wall fiber degradation (pectin, xylan and cellulose) followed later by the slow degradation of starch and involving different microbial communities (90). Accessible intracellular material from broken cells also impacts the fermentation kinetics of plant cell walls, as demonstrated *in vitro* with differently processed wheat bran. A reduced SCFA production was found in wheat bran from which remaining starch fractions had been enzymatically removed (87). In the same experiment, the effect of fine milling was demonstrated with micronized (very finely milled) bran leading to higher initial SCFA production *in vitro* compared to unmodified bran. This observation had been made previously and attributed to a higher bacteria-to-surface ratio with smaller particle sizes (91). However, at the end of the fermentations, SCFA levels were similar (87, 91, 92), but the ratios between SCFA differed, with larger bran particles producing more butyrate (91, 92). This is particularly interesting since more extensive processing of bran, such as extrusion, is considered desirable as it makes the constituents more accessible for microbial fermentation by degrading the three-dimensional bran structure. However, this may reduce the potentially health-promoting butyrate production (57, 93, 94). In summary, the intrinsic structural features of the plant cells likely slow down fiber fermentation, inducing a lag phase (58), but do not necessarily reduce the absolute amount of SCFA produced. Consequently, there is a gradual release of SFCA, which means that SCFA production is not restricted to the proximal colon but spread throughout the whole colon, including its distal parts benefitting local, mucosal health. This likely translates into beneficial systemic, peripheral effects as distal SCFA infusion *in vivo* has shown to induce more pronounced effects on biomarkers than proximal (95). Also delayed fermentation has been proposed as a desired feature of fibers in the treatment of irritable bowel syndrome but lacks presently experimental verification (23). In summary, the delayed fermentation of intrinsic fibers presents a highly relevant feature that isolated, single fibers do not have.

### Insights from existing human studies

Whether the observed features of intrinsic fibers *in vitro* also occur *in vivo* and how these effects are reflected in health outcomes is not yet clear. To advance our insight, we summarized human intervention studies that investigated the effect of intrinsic fibers on gut microbiota composition and activity and/or related metabolic and bowel function outcomes (**Table 1**; extended version in **Supplementary Table S1**). We included randomized-controlled trials (no acute testing, patient-control or single-arm designs) published during the last 20 years. These trials either assessed diets based on whole foods or used one specific food (including vegetables, fruits and nuts) either in its fresh, cooked or dried form. If whole foods were further processed during the study to be incorporated into meals we did include those as well, but excluded pulverized versions of food products as the mechanical processing extensively destroys the plant matrix. Similarly, we excluded fruit and vegetable pastes, juices or extracts as these underwent extended processing. For each study we have presented the processing type based on the reported information as to understand the impact on the intrinsic fiber structure. We did not include studies using waste-stream products as the level of information on the applied processing is generally insufficient to decide whether intrinsic structures are maintained or not. Based on provided information on fold-changes in relative abundance of gut microbial taxa we estimated the modulatory potential to be either small (<1.5-fold), moderate (1.5 - 2.5-fold) or large (>2.5-fold), which is summarized in an extended version of **Table 1** (**Supplementary Table S1**).

**Table 1:** Human intrinsic fiber studies.

Intrinsic fiber	Processing	Study design	Gut microbiota composition	Microbiota activity	Metabolic markers	Bowel function	Reference
**Whole diets**							
Mediterranean diet	Whole foods; incorporated into meals @Home	RCT, 1 year, parallel (dietary advice & provided foods)	Yes ‡	–	Yes	–	(96, 97)
Nordic diet	Whole foods; incorporated into meals @Home	RCT, 18 or 24 weeks, parallel (dietary advice & provided foods)	Not assessed	–	Yes	–	(98, 99)
Macrobiotic diet	Whole foods; incorporated into meals by cooks	RCT, 3 weeks, parallel (controlled diet)	Yes ‡	–	Yes	–	(100)
**Bran**							
Wheat bran (20 g/day)	Coarse vs fine	RCT, 4 weeks, parallel	–	–	–	Yes	(101)
Wheat bran (12-22 g/day)	Raw vs cooked	RCT, 2 weeks, cross-over	–	–	–	Yes (raw)	(102)
Wheat bran (20 g/day)	Reduced in size	RCT, 4 weeks, parallel (normal/obese)	No change	No change	Yes (obese)	No change	(103)
**Grains**							
Barley (75 g/day)	Whole kernels, boiled, in bread (no milling)	RCT, 4 weeks, cross-over	–	–	Yes	–	(104)
Barley vs brown rice vs mix of both (60 g/day)	Whole kernels, cooked	RCT, 28 days, cross-over	Yes, moderate Δfold	–	Yes (mix)	–	(105)
Coix (160 g/day)	Whole kernels, cooked	RCT, 1 week, parallel	Yes, small Δfold	–	Yes	–	(106)
**Nuts**							
Walnut (42 g/day)	Whole	RCT, 3 weeks, cross-over	Yes, moderate changes	–	Yes	–	(107)
Almond (57 g/day)	Whole, roasted	RCT, 6 weeks, parallel	Yes, small & large Δfold	–	-	–	(108)
Almond (42 g/day)	Whole raw, whole roasted, chopped roasted, almond butter	RCT, 3 weeks, cross-over	Yes, moderate to large Δfold (most chopped)	–	-	–	(109)
Almond or pistachio (43 or 85 g/day)	Whole	RCT, 2.5 weeks, cross-over	Yes ‡, stronger pistachio effect	–	–	–	(110)
**Legumes & Seeds**							
Chickpea or raffinose (200g vs 5 g/day)	Canned; incorporated into soups & desserts	RCT, 3 weeks, cross-over	Yes ‡	No change	–	–	(111)
Linseed, sunflower & sesame seed, wheat grain, haricot & kidney bean, chickpea	Whole vs ground; incorporated into meals (no milling)	RCT, 1 week, cross-over, (controlled diet)	–	Yes	–	Yes	(112)
**Vegetables**							
Broccoli, cauliflower ^+^/_-_ green & red cabbage(~ 800 g/day)	Raw and incorporated in soup or microwaved	RCT, 2 weeks, cross-over (controlled diet)	Yes ‡	–	–	–	(113)
Broccoli and cauliflower (168 ^+^/_-_ 300 soup g/day)	Frozen & steamed or incorporated into soup	RCT, 2 weeks, cross-over	Yes ‡	–	–	–	(114)
Chicory root (30 g/day)	Dried, cut into cubes (3 g)	RCT, 3 weeks, parallel	Yes, large Δfold	Yes	Yes	Yes	(115)
**Fruits**							
Avocado (1 piece/day)	Wholefood	RCT, 12-weeks, parallel (hypocaloric diet)	Yes, moderate to large Δfold	–	Yes	–	(116)
Avocado (140-175 g/day)	Whole food, part of meal	RCT, 12 weeks, parallel (partly controlled diet)	Yes, small Δfold	Yes	Yes	–	(117)
Mango (300 g/day)	Whole food	RCT, 4 weeks, parallel	–	Yes	Yes	Yes	(118)
Kiwi (2 pieces/day)	Whole food	RCT, 3 days, cross-over	–	–	–	Yes	(119)
Date (~50 g/day)	Dried	RCT, 3 weeks, cross-over	No change	Yes	–	Yes	(120)
Prune (80 or 120 g/day)	Dried	RCT, 4 weeks, parallel	Yes, small Δfold	No change	–	Yes	(121)
Raisin (120 g/day)	Dried	RCT, 3 weeks, cross-over	–	Yes	–	Yes	(122)

RCT, randomized-controlled trials; Δfold, fold changes in relative abundances; ‡ no information on fold changes provided. We selected randomized-controlled trails that have either used diets or single whole foods to assess their modulatory potential on gut microbiota and/or related health effects. These trials were published during the past 20 years, except for two of the bran studies. Even though bran does not necessarily classify as intrinsic fibers (depending on its processing), we did include some old and recent bran studies due to the long and ongoing interest in this type of plant food. Details of the trials are provided in the **Supplemental Table S1**.

Whole food interventions are particularly relevant in the context of intrinsic fibers as subjects do not consume one but a variety of differently processed intrinsic fibers. Most of these studies (**Table 1**, Diets) have in common that they emphasize whole grains, fruits and vegetables often combined with legumes and nuts (96), specific types of grains and berries (98) as well as dairy and animal products, while others can be more restricted (macrobiotic diet) (100). These diets have been linked to numerous beneficial health outcomes, like improvements in markers of cognitive function (97), inflammation (97), lipid (98–100) and glucose metabolism (98, 100), and in subjects adhering to these diets these changes were associated with increased levels of gut microbial taxa involved in fiber breakdown and short-chain fatty acid production (e.g. *Faecalibacterium* spp.; **Supplementary Table S1**).

Although strictly speaking wheat bran is not necessarily an intrinsic fiber as its intactness largely depends on the degree of processing, we also included old and recent wheat bran studies (**Table 1**, Bran). Bran has been investigated in the 1970’s and 1980’s for its modulatory effect on lipid metabolism and bowel function and current approaches potentially provide insights into underlying gut microbiota modulation. Especially the early studies assessed processing effects, like different milling degrees and concluded that only coarse bran reduced transit time and intraluminal pressure (**Supplementary Table S1**) (101). Also hydrothermal processing (cooking) impaired effectiveness of wheat bran and only raw but not cooked bran influenced bowel function (**Supplementary Table S1**) (102). Surprisingly, with the available high-throughput technologies only very limited effects of bran on gut microbiota composition and activity were observed in a recent study with bran of different particle sizes (**Supplementary Table S1**) (103). However, also in bread-based interventions and acute settings few effects are found (123, 124), likely linked to the applied processing (fine milling, and enzymatic treatment). Moreover, bran and enclosed starch might have a synergistic function in the whole kernel, as in isolation they have been found to distinctly and differently impact gut microbiota (125, 126). For instance, intake of resistant starch increased the relative levels of *Ruminococcaeae* and decreased members of the *Lachnospiraceae* family, while wheat bran induced the opposite effect (125, 126). In contrast, two whole-kernel barely interventions with or without brown rice (104, 105) and a coix seed intervention (106) did modulate gut microbiota response by increasing health-associated fiber-degraders such as *Bifidobacterium, Roseburia* or *Faecalibacterium* spp. and further impacted metabolic outcomes, like decreasing inflammatory markers (**Table 1**, Grains; **Supplementary Table S1**). This indicates that the whole kernel of grains likely acts differently than the separated, milled and reconstituted kernel fractions in whole-grain products. Moreover, this confirms the notion described above that whole grain products do not necessarily provide intrinsic fibers (35, 67).

Nuts are another increasingly studied source of intrinsic fibers as their plant cells are filled with lipid bodies (**Figure 1B**). Information on lipid-degrading gut bacteria is limited and, generally, the amount of fat reaching the colon is believed to be low as most fats we consume are of animal origin or extracted from plants (oil) (127). Lipid bodies in nuts, however, resist upper gut digestion (77, 128, 129). Consequently, studying the impact of nuts on the gut microbiota is of interest and a considerable number of studies assessed the modulatory potential of especially walnuts and almonds in various processed forms, as has been reviewed comprehensively (130). Indeed, nuts generally appear to exert moderate effects on various taxa, yet these effects are not consistent within and between nut types (**Supplementary Table S1**). One study assessed the effect of different almonds preparations and as expected their most processed form, which was almond butter, had only a small to moderate modulatory effect on the affected taxa when consumed (**Supplementary Table S1**) (109). In contrast, chopped and roasted almonds exerted the largest modulatory effect on the affected taxa (109). It is tempting to hypothesize that increased particles size improves but roasting impairs the efficiency of the intrinsic matrix disintegration in the upper gut (128, 129), which in turn impacts gut microbial breakdown. Unfortunately, these nut studies did generally not assess other parameters like fecal SCFA or bowel function (**Table 1**, Nuts).

To the best of our knowledge only one human study tried to assess the difference between isolated and intrinsic fiber structure in adult volunteers (111). In this study canned chickpeas were compared to raffinose (**Table 1**, Legumes & Seeds). While the intactness of the plant cell structure was likely already impaired in the canned chickpeas, we hypothesize that this was even further reduced by mechanical processing, as both fiber types were incorporated into soups and desserts. Both fibers slightly affected the gut microbiota composition compared to control, with the suggestion that chickpea induced a decrease of ammonium-producing strains (111). Another study assessed a variety of whole seeds, legumes and wheat grains mixed together and compared them against their ground form incorporated into meals (112). Both types of meals improved bowel function but only the meals with unground seeds, legumes and grains increased fecal butyrate as well as total SCFA (**Supplementary Table S1**) (112).

Vegetables have been rarely investigated in their intrinsic form and fruits are mainly assessed dried, but not fresh (**Table 1**, Vegetables). This might be due to the fact that more attention has been given to their phytochemicals, which require cell breakage to be released from the vacuole. Two studies assessing cooked cruciferous vegetables prepared in various dishes observed modulation of the gut microbiota composition, but did not indicate how affected taxa related to fecal SCFA levels and other outcomes (**Supplementary Table S1**) (113, 114). Recently, we reported on the intake of dried chicory root cubes (approximately 3 mm rib), as a palatable preparation of this root vegetable high in inulin-type fructans (115). The product had major effects on gut microbiota composition and activity, inducing a butyrogenic trophic chain including *Bifidobacterium* and *Anaerostipes* spp. and improved bowel function (**Supplementary Table S1**). Of note, fecal SCFA levels were highly increased to an extent never observed with isolated inulin. While the variation of blood glucose levels was reduced by the dried chicory root, fasting plasma markers were only slightly impacted, which we found to relate to baseline gut microbiota composition (115). Interestingly, a single-arm trial, using meals based on inulin-rich vegetables (e.g. onion, Jerusalem artichoke, leeks), did not observe changes in fecal SCFA levels despite a major increase in *Bifidobacterium* spp. (131). This could relate to the low dosage (~15 g/day fresh vegetable) and damage to cell walls by cooking, releasing encapsulated inulin already in the proximal colon.

There are some studies that investigated various effects of fresh fruits like avocado, mango and kiwi and dried fruits like prunes, raisin and dates (**Table 1**, Fruits). Similarly to nuts, avocados are plant foods rich in fat, but despite their high fat content have been found to decrease circulating triglyceride levels and concomitantly increase fecal fat and bile acid output (116, 117). While fiber can exert these positive effects on lipid metabolism by microbiota-independent effects and the small intestinal microbiota is known to impact bile acid metabolism (8, 132), the role of the colonic microbiota is not yet understood. As avocado intake was reported to increase levels of bacteria normally related to a diet high in fat from animal foods (e.g. *Bilophila* (133)) and associated with negative health outcomes, future studies will shed light onto the microbiota-mediated health benefits of these intrinsic fibers (**Supplementary Table S1**) (116, 117). Many other fruit studies have focused on the application of intrinsic fibers to stimulate bowel function, and for a comprehensive overview hereof we refer the reader to others (134). Unfortunately, the majority of these fruit studies did not address the gut microbiota and its products, such as butyrate and other SCFA (119, 135, 136). One recent study compared mango to an equal fiber dose of psyllium (**Supplementary Table S1**) (118). Psyllium likely relieves constipation by microbiota-independent effects as only a minimal impact on gut microbiota composition and SCFA production has been reported (137). In contrast, the mango fruit improved bowel function and also increased fecal SCFA and decreased IL-6 levels (**Supplementary Table S1**) (118). Also intake of dried raisins improved bowel function and increased fecal SCFA (**Supplementary Table S1**) (122). These results suggests that (dried) fruits that are metabolizable by the gut microbiota in the colon have microbiota-dependent health impacts related to their intrinsic fibers.

Overall, human intervention studies assessing intrinsic fibers confirm that these fibers impact the gut microbiota in various ways despite the possible physical barrier and complexity of the plant cell matrix. The majority of the reported effects on gut microbiota composition and activity is small to moderate, with exception of the dried chicory root particles that had major effects (**Table 1**, Vegetables). Moreover, few studies assessed changes in gut microbiota composition and activity together with metabolic markers and bowel function, which makes the translation of the observed effects challenging. In future it is important to investigate the effect of processing including particle size of intrinsic fibers evaluated against isolated, single fibers.

## Conclusions and considerations for future research

Modulating the gut microbiota using dietary fibers is an exciting field likely resulting in new therapeutic avenues to maintain and improve human health. Research on fiber-microbiota interactions has followed for years a reductionist approach based on the concept that isolated fibers are needed to understand how dietary fibers impact human health. However, during digestion dietary fibers do not simply dissolve from the plant tissues making them available to the gut microbiota as single components. Instead, plant tissue fractions are maintained and their complexly intertwined cell walls and encapsulated fructans and starch polymers arrive in the colon. These intrinsic fibers likely slow down colonic bacterial fermentation as the gut bacteria cannot spatially access all cell wall fibers and encapsulated contents. Thereby fiber-derived SCFA production is likely spread throughout the entire length of the colon, notably the distal colon with described health benefits (95, 138). However, how these processes evolve is barely understood. Research assessing intact plant tissue fractions and cells has focused mainly on the upper gut. Few studies have assessed the further breakdown of intrinsic fibers in the colon and are mainly based on *in vitro* or animal *in vivo* data. Hence, there is a clear lack of human *in vivo* data on the utilization of intrinsic fibers by the gut microbiota. Future research should focus on understanding (i) how intrinsic fiber structures differ from isolated single fibers in their fermentation kinetics, (ii) how the gut microbiota spatially colonizes intrinsic fiber particles and cooperates with other bacteria in the liquid and mucosal environment, and (iii) how intrinsic fibers from different plant sources and their processing affects microbial breakdown and related human health outcomes. Finally, with the shift from animal-based to more sustainable, minimally-processed plant-based diets we should put considerable effort in the understanding how plant cell-encapsulated proteins and fats affect the gut microbiota in the distal colon in contrast to animal-derived equivalents.

Food processing can fundamentally affect the intrinsic fiber structure (**Figure 1C**). Elucidating how different domestic preparation techniques (e.g. raw versus cooked in water versus steamed vegetables) affect health status by modulating the gut microbiota is an exciting field that has rarely been explored (75, 139). In this context it is also important to be reminded that food processing per se is not health-detrimental. Certain foods are barely digestible without any processing and for specific populations, e.g. those suffering from malnutrition or diseases, food processing is crucial. However, in the Western population that consumes an abundance of highly (over)processed foods, the increased digestibility has resulted in negative health outcomes related to obesity and welfare diseases (140). Unsurprisingly, focusing on assessing the isolated effects of fibers and relying on them to create new food designs stimulates food processing and the production of waste streams but not necessarily promotes the development of healthy foods. As fibers in their unextracted, minimally processed form of the intrinsic plant cell matrix provide health benefits by naturally encapsulating ingredients and slowing down dietary fiber fermentation, we need to rethink the way we use dietary fibers in healthy food design. Hence, we postulate that future food designs should rather reduce the extent of food processing and move towards exploiting the intrinsic plant cell matrix, which we find in any plant food (**Figures 1A–C**). By doing so, we might not only reduce the level of food processing, but also reduce waste and create new healthy products that are in line with more sustainable and plant-based oriented diets.

## Author contributions

M-LP and WMdV conceptualization; M-LP literature search, writing initial draft; WMdV supervision, critical revision. All authors contributed to the article and approved the submitted version

## Funding

Part of this work was supported by the Innovation Program Microbiology of Wageningen University (IPM-4 2022, Small Science Seeds).

## Acknowledgments

We thank Frederik S. Kaper from WholeFiber BV for his technological insights on fiber structures and processing effects. We are also grateful to Prof. dr. Edith Feskens and Prof. dr. Hauke Smidt for the stimulating discussions.

## Conflict of interest

WV provided scientific advice to WholeFiber BV.

The remaining author declares that the research was conducted in the absence of any commercial or financial relationships that could be construed as a potential conflict of interest.

## Publisher’s note

All claims expressed in this article are solely those of the authors and do not necessarily represent those of their affiliated organizations, or those of the publisher, the editors and the reviewers. Any product that may be evaluated in this article, or claim that may be made by its manufacturer, is not guaranteed or endorsed by the publisher.

## Abbreviations

SCFA, short-chain fatty acids.
