# Supplementary material for: Intrinsic dietary fibers and the gut microbiome: Rediscovering the benefits of the plant cell matrix for human health
Source: Front Immunol. 2022 Aug 18;13:954845. doi: 10.3389/fimmu.2022.954845 (PMC9434118; doi:10.3389/fimmu.2022.954845)
Supplement: Supplementary file 1 [file DataSheet_1.pdf]

Supplementary Material

1 Supplementary Table S1

The following table is an extended version of Table 1 in the main article. We summarized existing human intervention trial that assessed intrinsic fiber in their relation to the gut microbiota and related health outcomes. Most of these fibers are in the form of whole foods, either as single foods or part of a whole food based diet. For this purpose, we selected randomized-controlled trials (excluding single-arm designs and patient-control designs) that were published during the last 20 year. The only exception is bran which - depending on its processing – is not necessarily an intrinsic fiber and has been studied extensively in the 1970's and 1980's. Hence, we included also bran studies older than 20 years. We did not include waste stream-derive fibers as the available information is mostly insufficient to make conclusion regarding the intactness of the intrinsic fiber structure.

Table S1 Dietary fiber interventions using whole foods. Intrinsic fibers have been rarely studied in human intervention trials. We selected human studies that have either used whole food diets or whole foods (fresh, cooked, dried) to assess their gut microbiota modulatory potential and related health effects. Even though bran does not necessarily classify as intrinsic fiber due to processing, we did include some old and recent bran studies here as bran has been widely tested in relation to human health and different processing conditions. If not indicated different the populations consist of healthy subjects.

Intrinsic fiber	Processing	Study design	Gut microbiota modulation (Δfold)	Changes in microbiota activity	Changes in metabolic markers	Changes in bowel function	Reference
**Whole diets**							
Mediterranean diet (whole grains, fruits & vegetables, legumes and nuts)	Whole foods; incorporated into meals @Home	RCT, 1 year, parallel (dietary advice & provided foods)	No clear Δfold reported, adherence associated with ↑ *Faecalibacterium, Roseburia*, *Eubacterium* ↓ *Ruminococcus, Dorea Colinsella, Coprococcus*	-	Association with ↑ Cognitive function ↓ CRP, IL-17	-	(1,2)
Nordic diet (rye, barley, oats, berries, fruit, vegetable)	Whole foods; incorporated into meals @Home	RCT, 18 or 24 weeks, parallel (dietary advice & provided foods)	-	-	↓ cholesterol markers, association with lipid, glucose metabolism	-	(3,4)
Macrobiotic diet (mainly vegetable & grains)	Whole foods; incorporated into meals by cooks	RCT,3 weeks, parallel (fully controlled)	No clear Δfold reported	-	↓ fasting & post-prandial glucose, triglyceride, cholesterol	-	(5)
**Bran**							
Wheat bran (20 g/day)	Coarse vs fine	RCT, 4 weeks, parallel	-	-	-	↑ colonic motility, ↓ transit time	(6)
Wheat bran (12 & 20 vs 13.2 & 22 g/day)	Raw vs cooked	RCT, 2 weeks, cross-over	-	-	-	↑ fecal weight, stool volume ↓ transit time (raw bran)	(7)
Wheat bran (20 g/day)	Reduced in size	RCT, 4 weeks, parallel (normal weight and obese)	No change	No change	↑ fasting serum acetate, total SCFA (obese subjects)	No change	(8)

-, not assessed; Δfold, fold-change in relative abundance; ↑, increase; ↓, decrease; +/-, with or without; CRP, C-reactive protein;, IL, interleukin; RCT, randomized-controlled trial, SCFA, short-chain fatty acids

Intrinsic fiber	Processing	Study design	Gut microbiota modulation (Δfold)	Changes in microbiota activity	Changes in metabolic markers	Changes in bowel function	Reference
**Grains**							
Barley (75 g/day)	Whole kernels, boiled, in bread (no milling)	RCT, 4 weeks, cross-over	-	-	↓ postprandial glucose, GLP-1, breath hydrogen	-	(9)
Barley vs brown rice vs mix of both (60 g/day)	Whole kernels, cooked	RCT, 28 days, cross-over	↑ α-diversity; Moderate Δfold: *Bacteroides*: 0.7-0.8 *Blautia*: 1.4-1.5 *Roseburia:*0.9-1.5 *Bifidobacterium:* 1.0-2.0	-	↓ IL-6 (mix)	-	(10)
Coix (160 g/day)	Whole kernels, cooked	RCT, 1 week, parallel	↓ α-diversity; Small Δfold: *Faecalibacterium:* 1.4	-	↑ & ↓ in subset of lymphocytes	-	(11)
**Nuts**							
Walnuts (42 g/day)	Whole	RCT, 3 weeks, cross-over	Moderate Δfold: *Ruminococcus*: 0.8; *Dorea*: 0.8; *Roseburia*: 1.7	-	↓ fecal bile acids, cholesterol	-	(12)
Almonds (57 g/day)	Whole, roasted	RCT, 6 weeks, parallel	↑ α-diversity, Small & Large Δfold: *Mollicutes*: 1.5; *Alistipes:* 0.6; *Sutterella*: 3.7; *Bacteroides fragilis*: 1.2	-	-	-	(13)
Almonds (42 g/day)	Whole raw (**WR**), whole roasted (**RO**), chopped roasted (**C**), almond butter (**B**)	RCT, 3 weeks, cross-over	Moderate to large Δfold: *Roseburia*: 1.0-1.8 (**B<RO/WR<C**) *Lachnospira*: 1.1-1.6 (**B<WR <RO<C**) *Dialister*: 1.0-2.6 (**B<C<RO<WR**) *Oscillospira*:1.0-1.4 (**WR<RO<B<C**) **(order of Δfold indicated per processing type as abbreviated)**	-	-	-	(14)
Almonds or pistachios (43 or 85 g/day)	Whole	RCT, 2.5 weeks, cross-over	No clear Δfold reported for stronger pistachio effect	-	-	-	(15)
**Legumes & Seeds**							
Chickpeas or raffinose (200 vs 5 g/day	Canned; incorporated into soups & desserts	RCT, 3 weeks, cross-over	No clear Δfold reported	No change	-	-	(16)
Linseeds, sunflower & sesame seeds, wheat grains, haricot & kidney beans, chickpeas	Whole vs ground; incorporated into meals (no milling)	RCT, 1 week, cross-over, (fully controlled diet)	-	↑ fecal butyrate, total SCFA ↓ fecal pH	-	↑ stool weight (whole & ground)	(17)

-, not assessed; Δfold, fold-change in relative abundance; ↑, increase; ↓, decrease; +/-, with or without; IL, interleukin; RCT, randomized-controlled trial, SCFA, short-chain fatty acids

Intrinsic fiber	Processing	Study design	Gut microbiota modulation (Δfold)	Changes in microbiota activity	Changes in metabolic markers	Changes in bowel function	Reference
**Vegetables**							
Broccoli, cauliflower +/- green & red cabbage (up to 800 g/day)	Raw and incorporated in soup or microwaved	RCT, 2 weeks, cross-over (controlled diet)	No clear Δfold reported, association with ↑ *Eubacterium, Egerthella, Alistipes Phascolarctobacterium, Burkholderiales*	-	-	-	(18)
Broccoli and Cauliflower (168 +/- 300 soup g/day)	Frozen & steamed or incorporated into soup	RCT, 2 weeks, cross-over	No clear Δfold reported for ↓ *Rikenellaceae, Ruminococcaceae, Mogibacteriaceae, Clostridium, Clostridiales*	-	-	-	(19)
Chicory root (30 g/day)	Dried, cut into cubes (~3mm)	RCT, 3 weeks, parallel	↑ β-diversity, large Δfold: *Bifidobacterium:* 4.1 & *Anaerostipes:* 3.2, trophic chain proof	↑ fecal acetate, propionate, butyrate	↓ HOMA-ir glucose variability, fasting glucose microbiota dependent	↑ stool frequency, consistency	(20)
**Fruits**							
Avocado (1 piece/day)	Wholefood	RCT, 12-weeks, parallel (hypocaloric diet)	Moderate to large Δfold: *Bacteroides:* 1.4; *Dialister:* 1.4; *Sutterella*: 3.4; *Bilophila*: 3.5	-	↓ triglyceride levels, CRP, IL-1	-	(21)
Avocado (140-175 g/day)	Whole food, part of meal	RCT, 12 weeks, parallel (partly controlled diet)	Small Δfold: *Ruminococcus*: 0.7, *Faecalibacterium*: 1.3-fold, *Rosburia:* 0.7, *Lachnospira:* 1.4	↑ fecal acetate	↓ fecal bile acids, ↑ fecal fatty acids	-	(22)
Mango (300 g/day)	Whole food	RCT, 4 weeks, parallel	-	↑ fecal valerate ↓ fecal endotoxins	↓ IL-6	↑ stool frequency, consistency	(23)
Kiwi (2 pieces/day)	Whole food	RCT, 3 days, cross-over	-	-	-	↑ stool volume, consistency, frequency	(24)
Dates (~50 g/day)	Dried	RCT, 3 weeks, cross-over	No change	↓ fecal ammonium	-	↑ stool frequency, consistency	(25)
Prunes (80 or 120 g/day)	Dried	RCT, 4 weeks, parallel	Small Δfold: *Bifidobacterium*: 1.0-1.1	No change	-	↑ stool weight, frequency	(26)
Raisin (120 g/day)	Dried	RCT, 3 weeks, cross-over	-	↑ fecal total SCFA, acetate, butyrate, propionate ↓ fecal bile acids	-	↑ stool consistency ↓ transit time	(27)

-, not assessed; Δfold, fold-change in relative abundance; ↑, increase; ↓, decrease; +/-, with or without; CRP, C-reactive protein; HOMA-ir, Homeostatic Model Assessment for Insulin Resistance, IL, interleukin; RCT, randomized-controlled trial, SCFA, short-chain fatty acid

References

1. Ghosh TS, Rampelli S, Jeffery IB, Santoro A, Neto M, Capri M, Giampieri E, Jennings A, Candela M, Turroni S, et al. Mediterranean diet intervention alters the gut microbiome in older people reducing frailty and improving health status: the NU-AGE 1-year dietary intervention across five European countries. *Gut* (2020) **69**:1218–1228. doi: 10.1136/GUTJNL-2019-319654
2. Berendsen A, Santoro A, Pini E, Cevenini E, Ostan R, Pietruszka B, Rolf K, Cano N, Caille A, Lyon-Belgy N, et al. A parallel randomized trial on the effect of a healthful diet on inflammageing and its consequences in European elderly people: Design of the NU-AGE dietary intervention study. *Mech Ageing Dev* (2013) **134**:523–530. doi: 10.1016/J.MAD.2013.10.002
3. Uusitupa M, Hermansen K, Savolainen MJ, Schwab U, Kolehmainen M, Brader L, Mortensen LS, Cloetens L, Johansson-Persson A, Önning G, et al. Effects of an isocaloric healthy Nordic diet on insulin sensitivity, lipid profile and inflammation markers in metabolic syndrome – a randomized study (SYSDIET). *J Intern Med* (2013) **274**:52–66. doi: 10.1111/JOIM.12044
4. Gürdeniz G, Uusitupa M, Hermansen K, Savolainen MJ, Schwab U, Kolehmainen M, Brader L, Cloetens L, Herzig KH, Hukkanen J, et al. Analysis of the SYSDIET Healthy Nordic Diet randomized trial based on metabolic profiling reveal beneficial effects on glucose metabolism and blood lipids. *Clin Nutr* (2022) **41**:441–451. doi: 10.1016/j.clnu.2021.12.031
5. Candela M, Biagi E, Soverini M, Consolandi C, Quercia S, Severgnini M, Peano C, Turroni S, Rampelli S, Pozzilli P, et al. Modulation of gut microbiota dysbioses in type 2 diabetic patients by macrobiotic Ma-Pi 2 diet. *Br J Nutr* (2016) **116**:80–93. doi: 10.1017/S0007114516001045
6. Kirwan W 0, Smith AN, Mcconnell AA, Mitchell WD, Eastwood MA. Action of different bran preparations on colonic function. *Br Med J* (1972) **4**:187–189. doi: 10.1136/bmj.4.5938.187
7. Wyman JB, Heaton KW, Manning AP, Wicks ACB. The effect on intestinal transit and the feces of raw and cooked bran in different doses. *Am J Clin Nutr* (1976) **29**:1474–1479. doi: 10.1093/AJCN/29.12.1474
8. Deroover L, Vázquez-Castellanos JF, Vandermeulen G, Luypaerts A, Raes J, Courtin CM, Verbeke K. Wheat bran with reduced particle size increases serum SCFAs in obese subjects without improving health parameters compared with a maltodextrin placebo. *Am J Clin Nutr* (2021) **114**:1328–1341. doi: 10.1093/ajcn/nqab196
9. Nilsson A, Johansson-Boll E, Sandberg J, Björck I. Gut microbiota mediated benefits of barley kernel products on metabolism, gut hormones, and inflammatory markers as affected by co-ingestion of commercially available probiotics: a randomized controlled study in healthy subjects. *Clin Nutr ESPEN* (2016) **15**:49–56. doi: 10.1016/J.CLNESP.2016.06.006
10. Martínez I, Lattimer JM, Hubach KL, Case JA, Yang J, Weber CG, Louk JA, Rose DJ, Kyureghian G, Peterson DA, et al. Gut microbiome composition is linked to whole grain-induced immunological improvements. *ISME J* (2013) **7**:269–280. doi: 10.1038/ismej.2012.104
11. Jinnouchi M, Miyahara T, Suzuki Y. Coix seed consumption affects the gut microbiota and the peripheral lymphocyte subset profiles of healthy male adults. *Nutrients* (2021) **13**: doi: 10.3390/NU13114079/S1
12. Holscher HD, Guetterman HM, Swanson KS, An R, Matthan NR, Lichtenstein AH, Novotny JA, Baer DJ. Walnut consumption alters the gastrointestinal microbiota, microbially derived secondary bile acids, and health markers in healthy adults: a randomized controlled trial. *J Nutr* (2018) **148**:861–867. doi: 10.1093/JN/NXY004
13. Dhillon J, Li Z, Ortiz RM. Almond snacking for 8 wk increases alpha-diversity of the gastrointestinal microbiome and decreases bacteroides fragilis abundance compared with an isocaloric snack in college freshmen. *Curr Dev Nutr* (2019) **3**: doi: 10.1093/CDN/NZZ079
14. Holscher HD, Taylor AM, Swanson KS, Novotny JA, Baer DJ. Almond Consumption and Processing Affects the Composition of the Gastrointestinal Microbiota of Healthy Adult Men and Women: A Randomized Controlled Trial. *Nutr 2018, Vol 10, Page 126* (2018) **10**:126. doi: 10.3390/NU10020126
15. Ukhanova M, Wang X, Baer DJ, Novotny JA, Fredborg M, Mai V. Effects of almond and pistachio consumption on gut microbiota composition in a randomised cross-over human feeding study. *Br J Nutr* (2014) **111**:2146–2152. doi: 10.1017/S0007114514000385
16. Fernando WMU, Hill JE, Zello GA, Tyler RT, Dahl WJ, Van Kessel AG. Diets supplemented with chickpea or its main oligosaccharide component raffinose modify faecal microbial composition in healthy adults. *Benef Microbes* (2010) **1**:197–207. doi: 10.3920/BM2009.0027
17. Hovey AL, Jones GP, Devereux HM, Walker KZ. Whole cereal and legume seeds increase faecal short chain fatty acids compared to ground seeds. *Asia Pacific J Clin Nutr* (2003) **12**:477–482.
18. Li F, Hullar MAJ, Schwarz Y, Lampe JW. Human gut bacterial communities are altered by addition of cruciferous vegetables to a controlled fruit- and vegetable-free diet. *J Nutr* (2009) **139**:1685. doi: 10.3945/JN.109.108191
19. Kellingray L, Tapp HS, Saha S, Doleman JF, Narbad A, Mithen RF. Consumption of a diet rich in Brassica vegetables is associated with a reduced abundance of sulphate-reducing bacteria: A randomised crossover study. *Mol Nutr Food Res* (2017) **61**:1600992. doi: 10.1002/MNFR.201600992
20. Puhlmann M-L, Jokela R, Van Dongen KCW, Bui TPN, Van Hangelbroek RWJ, Smidt H, De Vos WM, Feskens EJM. Dried chicory root improves bowel function, benefits intestinal microbial trophic chains and increases faecal and circulating short chain fatty acids in subjects at risk for type 2 diabetes. *Gut Microbiome* (2022) **3**:e4. doi: 10.1017/GMB.2022.4
21. Henning SM, Yang J, Woo SL, Lee RP, Huang J, Rasmusen A, Carpenter CL, Thames G, Gilbuena I, Tseng CH, et al. Hass avocado inclusion in a weight-loss diet supported weight loss and altered gut microbiota: a 12-week randomized, parallel-controlled trial. *Curr Dev Nutr* (2019) **3**:1–9. doi: 10.1093/CDN/NZZ068
22. Thompson S V., Bailey MA, Taylor AM, Kaczmarek JL, Mysonhimer AR, Edwards CG, Reeser GE, Burd NA, Khan NA, Holscher HD. Avocado consumption alters gastrointestinal bacteria abundance and microbial metabolite concentrations among adults with overweight or obesity: a randomized controlled trial. *J Nutr* (2021) **151**:753–762. doi: 10.1093/JN/NXAA219
23. Venancio VP, Kim H, Sirven MA, Tekwe CD, Honvoh G, Talcott ST, Mertens-Talcott SU. Polyphenol-rich mango (Mangifera indica L.) ameliorate functional constipation symptoms in humans beyond equivalent amount of fiber. *Mol Nutr Food Res* (2018) **62**: doi: 10.1002/MNFR.201701034
24. Wilkinson-Smith V, Dellschaft N, Ansell J, Hoad | Caroline, Marciani L, Gowland | Penny, Spiller R. Mechanisms underlying effects of kiwifruit on intestinal function shown by MRI in healthy volunteers Summary Background: Chronic constipation affects approximately 17% of the population. *Aliment Pharmacol Ther* (2019) **49**:759– 768. doi: 10.1111/apt.15127
25. Eid N, Osmanova H, Natchez C, Walton G, Costabile A, Gibson G, Rowland I, Spencer JPE. Impact of palm date consumption on microbiota growth and large intestinal health: a randomised, controlled, cross-over, human intervention study. *Br J ofNutrition (2015),* (2015) **114**:1226–1236. doi: 10.1017/S0007114515002780
26. Lever E, Scott SM, Louis P, Emery PW, Whelan K. The effect of prunes on stool output, gut transit time and gastrointestinal microbiota: A randomised controlled trial. *Clin Nutr* (2019) **38**:165–173. doi: 10.1016/J.CLNU.2018.01.003
27. Spiller GA, Story JA, Furumoto EJ, Chezem JC, Spiller M. Effect of tartaric acid and dietary fibre from sun-dried raisins on colonic function

and on bile acid and volatile fatty acid excretion in healthy adults. *Br J Nutr* (2003) **90**:803–807. doi: 10.1079/BJN2003966
